# Mandibuloacral dysplasia type B (MADB): a cohort of eight patients from Suriname with a homozygous founder mutation in *ZMPSTE24* (*FACE1*), clinical diagnostic criteria and management guidelines

**DOI:** 10.1186/s13023-019-1269-0

**Published:** 2019-12-19

**Authors:** M. M. Hitzert, S. N. van der Crabben, G. Baldewsingh, H. K. Ploos van Amstel, A. van den Wijngaard, C. M. A. van Ravenswaaij-Arts, C. W. R. Zijlmans

**Affiliations:** 1 Department of Genetics, University of Groningen, University Medical Center Groningen, Groningen, The Netherlands; 2 Department of Medical Genetics, Amsterdam UMC, University of Amsterdam, Amsterdam, The Netherlands; 3 Medical Mission Primary Health Care Suriname, Paramaribo, Suriname; 4 Department of Clinical Genetics, Maastricht University Medical Center, Maastricht, The Netherlands; 5 Scientific Research Centre Suriname, Academic Hospital Paramaribo, Paramaribo, Suriname; 6 Faculty of Medical Sciences, Anton de Kom University of Suriname, Paramaribo, Suriname; 7 Department of Paediatrics, Diakonessenhuis Hospital, Paramaribo, Suriname

**Keywords:** Mandibuloacral dysplasia with type B lipodystrophy, *ZMPSTE24* gene, Suriname, Diagnostic criteria

## Abstract

**Background:**

Mandibuloacral Dysplasia with type B lipodystrophy (MADB) is a rare premature aging disorder with an autosomal recessive inheritance pattern. MADB is characterized by brittle hair, mottled, atrophic skin, generalized lipodystrophy, insulin resistance, metabolic complications and skeletal features like stunted growth, mandibular and clavicular hypoplasia and acro-osteolysis of the distal phalanges. MADB is caused by reduced activity of the enzyme zinc metalloprotease ZMPSTE24 resulting from compound heterozygous or homozygous mutations in *ZMPSTE24*.

**Methods:**

In 2012, and again in 2018, eight related patients from the remote tropical rainforest of inland Suriname were analysed for dysmorphic features. DNA analysis was performed and clinical features were documented. We also analysed all previously reported genetically confirmed MADB patients from literature (*n* = 12) for their clinical features. Based on the features of all cases (*n* = 20) we defined major criteria as those present in 85–100% of all MADB patients and minor criteria as those present in 70–84% of patients.

**Results:**

All the Surinamese patients are of African descent and share the same homozygous c.1196A > G, p.(Tyr399Cys) missense variant in the *ZMPSTE24* gene, confirming MADB. Major criteria were found to be: short stature, clavicular hypoplasia, delayed closure of cranial sutures, high palate, mandibular hypoplasia, dental crowding, acro-osteolysis of the distal phalanges, hypoplastic nails, brittle and/or sparse hair, mottled pigmentation, atrophic and sclerodermic skin, and calcified skin nodules. Minor criteria were (generalized or partial) lipoatrophy of the extremities, joint contractures and shortened phalanges. Based on our detailed clinical observations, and a review of previously described cases, we propose that the clinical diagnosis of MADB is highly likely if a patient exhibits ≥4 major clinical criteria OR ≥ 3 major clinical criteria and ≥ 2 minor clinical criteria.

**Conclusions:**

We report on eight related Surinamese patients with MADB due to a homozygous founder mutation in *ZMPSTE24*. In low-income countries laboratory facilities for molecular genetic testing are scarce or lacking. However, because diagnosing MADB is essential for guiding clinical management and for family counselling, we defined clinical diagnostic criteria and suggest management guidelines.

## Introduction

Mandibuloacral Dysplasia with type B lipodystrophy (MADB) is a rare premature aging disorder (OMIM # 608612) with and autosomal recessive inheritance pattern. It is characterized by brittle or absent hair, mottled and atrophic skin, generalized lipodystrophy, insulin resistance, teeth abnormalities, metabolic complications and skeletal features like stunted growth, mandibular and clavicular hypoplasia and acro-osteolysis of the distal phalanges [1]. Renal [2–4], cardiac and muscle involvement [4] have been described in some patients. The first clinical signs of MADB may be apparent at birth [3]. MADB patients’ cognition and social behaviour are usually normal. Diagnosis of MADB is based on history, physical examination, body composition and metabolic status. As yet, no clinical diagnostic criteria have been established [5], although Haye and colleagues [6] considered failure of ossification of the interparietal region of the occipital bone as a possible pathognomonic sign for MADB. Molecular genetic testing can confirm a diagnosis of MADB in most cases.

MADB is caused by reduced activity of the enzyme zinc metalloprotease ZMPSTE24 as a result of compound heterozygous or homozygous mutations in *ZMPSTE24* located on chromosome 1p34. A normal level of zinc metalloprotease is necessary to cleave the prenatylated and carboxylated 15-amino acid tail from the C-terminus of prelamin A to yield mature lamin A. The decreased function of zinc metalloprotease *ZMPSTE24* leads to an abnormal accumulation of lamin A precursors. Mature lamin A, together with the B-type lamin and lamin C, is mandatory for providing the shape, structure and function of the nuclear lamina [7]. *ZMPSTE24* mutations leading to a complete loss of enzyme activity are associated with the lethal syndrome restricted dermopathy (RD, OMIM #275210) [8]. The more common Mandibuloacral Dysplasia with type A lipodystrophy (MADA; OMIM #248370) shares features with MADB, but is characterized by partial lipodystrophy of the extremities. MADA is caused by mutations in *LMNA* located on chromosome 1q22, leading to unprocessed lamin A [8].

To date, 12 genetically confirmed MADB patients have been reported in literature [1, 3, 4, 6, 9–14]. Here we present eight related patients of African descent from the remote tropical rainforest of inland Suriname with MADB caused by a homozygous pathogenic missense variant in *ZMPSTE24.* This variant has previously been reported in another patient with MADB, is located in a highly conserved area across species, predicted to be probably damaging (by prediction programmes such as SIFT and Polyphen) and has so far not been reported in gnomAD [6]. Our clinical description of MADB in this related family clearly depicts the phenotypic spectrum across different ages.

No clinical diagnostic criteria for MADB have been established thus far, but such criteria could be useful in remote areas in the absence of laboratory diagnostic possibilities. Establishing a diagnosis is essential for guiding clinical management and counselling patients and their family members. We therefore propose clinical diagnostic criteria and management guidelines based on the features found in our patients and previously reported MADB patients.

## Patients and methods

In 2012, and again in 2018, patients were referred to the clinical geneticist in the outpatient clinic for diagnostic work-up because of unexplained dysmorphic features and painful feet, which had been diagnosed previously by medical doctors of the Medical Mission Primary Health Care Suriname (MM).

### Patients

In 2012 the first six patients with dysmorphic features and painful feet were medically examined as part of the Medical Mission Primary Health Care Suriname program. The examination took place in an outpatient clinic in inland Suriname. Diagnostic genetic testing was performed after written informed consent from the patients or their legal representatives in accordance with local regulations. In 2018, the patients were invited to the department of Paediatrics of the Diakonnessenhuis hospital in Paramaribo for follow-up. For the calculation of standard deviations of height, weight for height and head circumference we used the growth curves of the TNO (Dutch organization for applied scientific research) [15].

Literature analysis of previous published patients was performed to establish phenotypic features. We systematically searched the PubMed database using the terms ‘Mandibuloacral dysplasia’ *AND* ‘ZMPSTE24’. We limited our search to full-text articles published in English and refined our search results by selecting only those articles that report on patients in whom MADB was genetically confirmed. We used the most recently published review article [16] to confirm that we had included all genetically confirmed MADB cases.

### Genetic testing

Genetic testing was performed in the Clinical Genetics laboratory of the University Medical Centre Utrecht. DNA was isolated from peripheral lymphocytes using standard procedures. First, the *LMNA* gene was sequenced in one patient and no likely pathogenic or pathogenic variant was identified. Hereafter, the *ZMPSTE24* gene was analysed, which revealed a homozygous missense variant that was classified as likely pathogenic by analysis in the Alamut Vision (v.2.11) mutation interpretation software program using e.g. SIFT, PolyPhen-2, GERP and Grantham scores and because of its absence in the Genome Aggregation Database (GnomADvs2.1). Subsequently, targeted mutation analysis was performed in the other affected family members.

### Phenotypic analyses

For each patient, the presence or absence of possible MADB features was documented. Possible MADB features were defined as clinical features noted more than once in MADB patients in the literature and more than once in the current cohort. The prevalence of each criterion was calculated by dividing the number of patients positive for that criterion by the number for whom that criterion was documented. In the absence of a gold standard for setting clinical criteria, we defined major criteria as clinical features present in 85–100% of all reported MADB patients. Minor criteria were defined as clinical features present in 70–84% of all reported MADB patients. Criteria that might be causally related, such as high palate, mandibular hypoplasia and dental crowding, were clustered. We also clustered hypoplastic nails and brittle or sparse hair since nail and hair abnormalities both represent ectodermic abnormalities. Skin abnormalities were clustered into one major criterion since all MADB patients showed two or more skin abnormalities. To determine cut-off points for establishing a clinical diagnosis of MADB we chose a combination of major and minor clinical features that applied to all MADB patients from our cohort and to most previously reported MADB patients.

Follow-up data were available for six patients, including blood glucose levels and blood pressure measurements. Based on the observations in our cohort we proposed management guidelines.

## Results

**Fig. 1:**
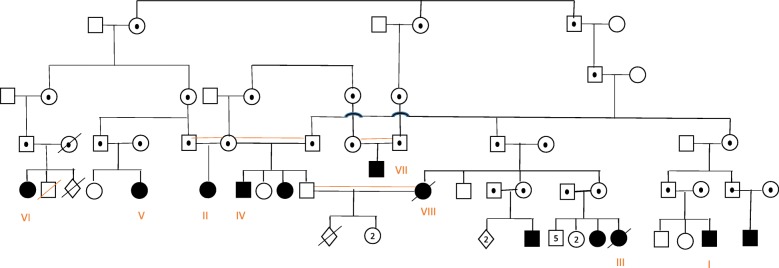
Pedigrees and pictures of the individuals studied. **a** Pedigree of the eight MADB patients suggestive of a common ancestor. Black boxes indicate affected individuals. White boxes with a dot are *presumed* heterozygous carriers of MADB. Roman numerals indicating the individual are plotted below each pedigree symbol

**Fig. 2:**
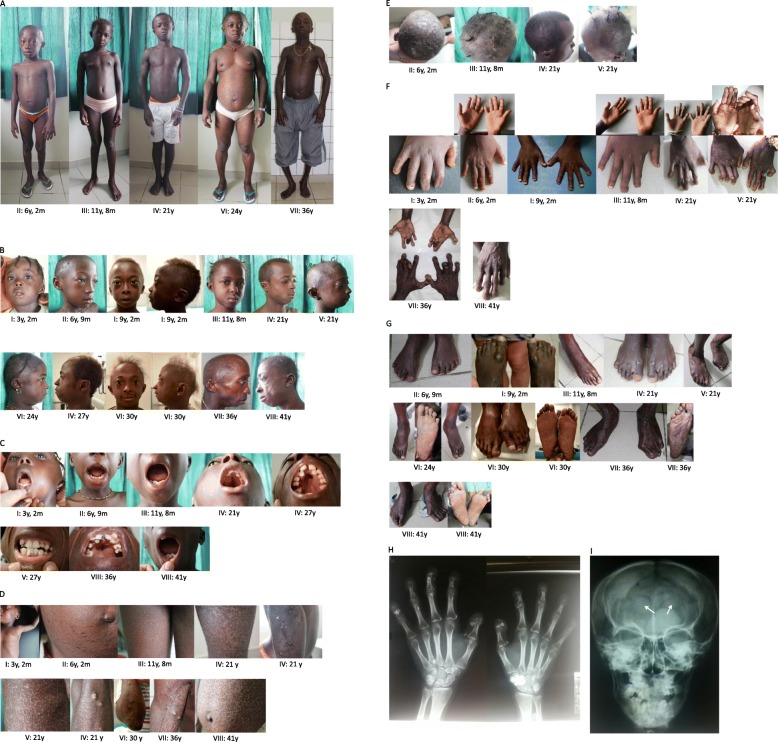
Images of individuals with MADB at chronological ages. Photographic images of **a** total body showing typical lipodystrophy; **b** facial features showing facial lipoatrophy, and mandibular hypoplasia; **c** oral region and teeth showing dental crowding, high palate; **d** skin showing calcified skin nodules and mottled, sclerodermic and atrophic skin in from left to right: patient, abdomen, legs (patient III, IV), arms (patients IV, V, IV, VI, VII) and abdomen **e** skull showing brittle and/or sparse hair; **f** hands and **g** feet showing acro-osteolysis of distal phalanges, shortened phalanges, joint contractures, hypoplastic nails, palmar and plantar atrophy of the hand and feet and calluses. X-rays of patient III showing **h** acro-osteolysis of the distal phalanges and **i** diminished occipital ossification (indicated by white arrows)

Sequence analysis of the *ZMPSTE24* gene revealed a previously documented [6] homozygous likely pathogenic mutation NM_005857.3: c.1196A > G, p.(Tyr399Cys) (OMIM#606480). Segregation of this variant was confirmed by targeted mutation analyses in seven other affected family members, thereby confirming its pathogenicity. A detailed clinical description of each patient is presented in Tables 1 and 2. A pedigree is shown in Fig. 1. See Fig. 2 for photographic images of the individual MADB patients.

**Table 1:** Patient characteristics at birth and at first examination

Patient characteristic	Patient I	Patient II	Patient III	Patient IV	Patient V	Patient VI	Patient VII	Patient VIII	Current cohort (*n* = 8)
Birth data									median (range)
Gender (male/female)	male	female	female	male	female	female	male	female	3/5
Birth weight (grams)	2880	2700	1830	2110	1830	1840	?	2180	2110 (1830–2880)
Birth weight (percentile)	p5-p15	p5-p15	<p1	<p1	<p1	<p1	?	<p1	
Height (cm)	45	48	43	43	?	41	?	?	43 (41–48)
Height (percentile)	<p1	p25-p50	<p1	<p1	?	<p1	?	?	
Head circumference (cm)	33	30	30	?	?	33	?	?	31.5 (30–33)
Head circumference (percentile)	p5-p15	<p1	<p1	?	?	p15-p25	?	?	
Growth parameters at 1st examination									
Age in years (y), months (m)	3y, 2 m	6y, 9 m	11y, 8 m	21y	21y	24y	36y	41y	21y (3–41)
Height (cm)	84	106.5	132	145	134	133	142	132	
Height-for-age (standard deviation)	−4.0	−3.0	−2.5	−5.0	−6.0	−6.2	−5.5	− 6.3	
Weight (kg)	?	15.3	26.8	40.6	28.4	39.1	32.2	34.1	
Weight-for-height (standard deviation)	?	−1.0	0.0	+ 1.8	+ 0.2	+ 2.5	0.0	+ 1.8	
Head circumference (cm)	45.4	49.5	48.5	54	50	52.8	52	48.5	
Head circumference-for-age (standard deviation)	−3.0	−1.0	−3.0	−2.0	−3.0	− 1.5	− 3.0	−3.7	

? = not assessed

**Table 2:** Overview of clinical features of patients with a homozygous or compound heterozygous mutation in the *ZMPSTE24* gene

Phenotypic features (yes/no)	Patient I	Patient II	Patient III	Patient IV	Patient V	Patient VI	Patient VII	Patient VIII	Current cohort (n = 8)	MADB patients from literature (*n* = 12)	total of MADB patients (*n* = 20)
Lipodystrophic features									*n/N (*percentage)	*n/N (*percentage*)*	*n/N (*percentage*)*
Lipoatrophy of the extremities	yes	yes	no	yes	yes	yes	yes	yes	7/8 (88)	7/11 (64)	14/19 (74)
Truncal lipoatrophy	yes	yes	no	no	yes	yes	yes	?	5/7 (71)	5/10 (50)	10/17 (59)
Generalized lipodystrophy	yes	yes	no	yes	yes	yes	yes	?	6/7 (86)	4/11 (36)	10/18 (56)
Facial lipoatrophy	yes	no	no	yes	yes	yes	yes	yes	6/8 (75)	4/12 (36)	10/20 (50)
Skeletal features											
Short stature	yes	yes	yes	yes	yes	yes	yes	yes	8/8 (100)	10/10 (100)	18/18 (100)
Underdeveloped occipital bone^a^	yes	yes	yes	yes	yes	yes	yes	yes	7/7 (100)	2/2 (100)	9/9 (100)
Hypoplastic nails	yes	yes	yes	yes	yes	yes	yes	yes	8/8 (100)	7/7 (100)	15/15 (100)
Acro-osteolysis of distal phalanges^b^	yes	yes	yes	yes	yes	yes	yes	yes	8/8 (100)	11/12 (92)	19/20 (95)
Delayed closure of cranial sutures	?	yes	?	yes	yes	yes	possible	?	5/5 (100)	9/10 (90)	14/15 (93)
Mandibular hypoplasia	yes	no	yes	yes	no	yes	yes	yes	6/8 (75)	12/12 (100)	18/20 (90)
Clavicular hypoplasia^c^	yes	yes	?	yes	yes	yes	?	yes	6/6 (100)	10/12 (83)	16/18 (89)
Shortened phalanges of the hands	yes	yes	yes	yes	yes	yes	yes	yes	8/8 (100)	7/11 (64)	15/19 (79)
Joint contractures	no	no	no	yes	yes	yes	yes	yes	5/8 (63)	9/12 (82)	14/20 (70)
Facial features											
High palate	yes	yes	?	yes	yes	yes	?	yes	6/6 (100)	3/3 (100)	9/9 (100)
Pinched nose	yes	no	no	no	no/bifid	yes	yes	?	3/7 (43)	9/11 (82)	12/18 (67)
Proptosis	no	no	no	yes	yes	yes	no	no	3/8 (38)	7/10 (70)	10/18 (56)
Posteriorly rotated ears	yes	no	no	no	yes	yes	yes	yes	5/8 (63)	3/7 (43)	8/15 (53)
Gaze palsy and/or ptosis	no	no	no	no	no	no	yes (ptosis)	no	1/8 (13)	1/4 (25)	2/12 (17)
Tooth abnormalities											
Dental crowding	yes	yes	yes	yes	yes	yes	yes	yes	8/8 (100)	6/7 (86)	14/15 (93)
Hypoplastic teeth	yes	no	?	no	no	yes	?	?	2/6 (33)	0/3 (0)	2/9 (22)
Hair abnormalities											
Brittle and/or sparse hair	yes	yes	yes	yes	yes	yes	yes	yes	8/8 (100)	9/12 (75)	17/20 (85)
Alopecia	no	no	no	yes	yes	yes	yes	yes	5/8 (50)	3/10 (33)	8/18 (44)
Skin abnormalities											
Mottled pigmentation	yes	yes	yes	yes	yes	yes	yes	yes	8/8 (100)	10/10 (100)	18/18 (100)
Skin atrophy	yes	yes	yes	yes	yes	yes	yes	yes	8/8 (100)	11/11 (100)	19/19 (100)
Sclerodermic skin	yes	yes	yes	yes	yes	yes	yes	yes	8/8 (100)	8/9 (89)	16/17 (94)
Calcified skin nodules	yes	yes	yes	yes	yes	yes	yes	yes	8/8 (100)	5/7 (71)	13/15 (87)
Normal psychomotor development	yes	yes	yes	yes	yes	yes	?	yes	7/7 (100)	7/9 (78)	14/16 (88)
Major / minor criteria (*n/n*)^#^	6/2	7/2	5/1	7/3	7/3	7/3	5/3	6/3			

? = not assessed. Short stature is defined as a length below the third percentile. No phenotypic data are available for two patients [8]. These patients are not included in this table

^a^based on physical examination: a palpable defect at the level of the occipital bone. For one patient (patient III), an X-ray of the skull was available

^b^ based on physical examination: presence of club-shaped digit(s) and/or prominent interphalangeal joints. For one patient (patient III), an X-ray of the hands was available

^c^ based on physical examination

^#^ see Table 3 for the definition of major and minor criteria

Patients ranged in age from 3 to 41 years. All patients from our cohort had a short stature, clavicular hypoplasia, delayed closure of cranial sutures, an underdeveloped occipital bone, acro-osteolysis of the distal phalanges, short phalanges of the hands, hypoplastic nails, a high palate, dental crowding, brittle and/or sparse hair and skin features (see Table 2 for details). Loss of subcutaneous fat from the palms of the hands and soles of the feet was noted in (nearly) all patients (see Table 2). The eldest patient had calcified nodules on the skin of her hands and arms. Patients IV, VII and VIII had developed contractures of the fingers and toes. All patients showed normal psychomotor development. Follow-up data are available for six patients (patients I, II, III, VI, VII and VIII) with patients III, IV and V lost to follow-up. Patient VIII died from heart failure at the age of 44 years. An X-ray demonstrated an enlarged heart in patient VIII and no additional cardiological evaluation was performed. None of the living patients developed diabetes mellitus. Patients VI and VII developed hypertension for which antihypertensive medication was started. Patient VII is suffering from dyspnoea and peripheral edema associated with his cardiac failure and will be evaluated by the cardiologist. Female patients did not report abnormalities of their period and were able to have normal reproduction (so far). See Table 2 for the clinical features of previously reported MADB patients.

Based on the prevalence of the clinical features as listed in Table 2, we defined major and minor clinical criteria (Table 3). We suggest that patients should exhibit ≥4 major clinical features OR ≥ 3 major clinical features and ≥ 2 minor clinical features in order to make the clinical diagnosis of MADB. All genetically proven MADB patients from our cohort fulfilled the diagnosis of MADB. In two out of the twelve MADB cases from the literature, an occipital ossification defect was reported [6, 12]. In the other ten MADB patients there was no information available on occipital ossification defects. All these ten MADB cases did exhibit ≥4 major clinical features OR ≥ 3 major clinical features.

**Table 3:** Proposed diagnostic criteria for MADB

Presence of ≥4 major clinical criteria OR presence of ≥3 major clinical criteria and ≥ 2 minor clinical criteria	
Major criteria^a^	Minor criteria^b^
- short stature (height < 2 standard deviations)	- (generalized or partial) lipoatrophy of the extremities
- clavicular hypoplasia	- joint contractures
- delayed closure of cranial sutures	- shortened phalanges
- high palate and/or mandibular hypoplasia and/or dental crowding	
- acro-osteolysis of distal phalanges (hands and/or feet)	
- hypoplastic nails and/or brittle or sparse hair	
- ≥2 of the following skin abnormalities:	
mottled pigmentation	
atrophic skin	
sclerodermic skin	
calcified skin nodules	

^a^ present in 85–100% of reported MADB patients

^b^ present in 70–84% of reported MADB patients

When we applied our clinical criteria to fourteen typical MADA patients with a proven *LMNA* mutation, they also fulfilled the criteria. The only discriminating feature between the MADA patients and the MADB patients is the occipital ossification defect, present in MADB patients only. One MADA patient (10 months old) exhibited only three major and one minor criteria and therefore did not fulfil the clinical criteria [17].

Once a diagnosis of MADB is considered, we recommend performing several evaluations based on the observations in our cohort (Table 4). Skeletal X-rays to evaluate for characteristic findings are not necessarily needed, but could guide in the diagnosis of MADB. Consultation with a clinical geneticist and/or genetic counsellor is recommended. When no genetic counsellor is available, the patient and/or the parents should be informed about the 25% risk of siblings of the patient having the same condition. Once the pathogenic variant has been identified in an affected family member, prenatal testing for a pregnancy at increased risk and preimplantation genetic diagnosis are possible (only available in some countries). Preventive measurements include a regular healthy diet and shoe pads, or at least comfortable footwear, because lack of body fat may lead to painful feet.

**Table 4:** Recommended evaluations in MADB patients

Evaluation	First evaluation	Frequency
Blood glucose level; evaluation of diabetes mellitus	following initial diagnosis	annually
Blood pressure measurement; evaluation of renal failure	following initial diagnosis	annually
Urine protein and glucose levels; evaluation of renal failure^a^	following initial diagnosis	annually
Podiatric evaluation; treatment of wounds due to plantar atrophy and/or peripheral neuropathy in diabetes mellitus patients	following initial diagnosis	annually
Dental examination; extraction of primary teeth may be required to avoid crowding and development of two rows of teeth	around the age of 4 years	annually
Blood lipid profiles; abnormal levels requires treatment including exercise, diet modification, and medication as warranted	around the age of 20 years	annually

^a^ This can be monitored by dipstick urine analysis

## Discussion

We describe eight related patients from remote inland Suriname with MADB due to the same homozygous likely pathogenic variant in the *ZMPSTE24* gene. In 2016, Haye and colleagues [6] described a male MADB patient carrying the same homozygous variant and that patient’s parents originated from the same tribal community as the patients in our cohort. It is therefore likely that Haye et al.’s patient shares the same ancestor as our patients, suggesting the existence of a founder mutation.

We classified the variant in our cohort as pathogenic based on prediction programmes (e.g. SIFT, PolyPhen), because of its absence in gnomADvs2.1, segregation of this variant in all affected family members, and because of clinical features that fit with the diagnosis of MADB.

The MADB patient described by Haye et al. [6] shows striking similarities to our patients: short stature, microcephaly, mandibular hypoplasia, sparse hair, mottled and atrophic skin, short phalanges of the hands with acro-osteolysis, delayed closure of cranial sutures and ossification failure of the occipital bone. Ossification failure of the occipital bone has also been observed in another MADB patient [12]. Haye et al. suggested that this feature might be a clinically diagnostic sign for MADB. In line with this, the occipital bone was underdeveloped in all of our patients. For one patient, an X-ray of the skull was available. In the other six patients a defect at the level of the occipital bone was clearly palpable. No ossification defect has been noted in other unaffected family members of this tribe. Our findings supports Haye et al.’s proposition that ossification failure of the occipital bone might be regarded an important clinical sign for MADB. Ossification defect of the occipital bone (without ossification defects of other cranial bones) is very uncommon and has not been linked to other diseases thus far. It has neither been linked to MADA patients, leading us to consider an occipital ossification defect as a pathognomonic feature distinguishing MADA from MADB patients. Based on the present literature we can, however, not exclude the presence of occipital ossification defects in MADA patients since radiographic examinations of the skull were only performed in six out of the fourteen MADA patients [10, 18–21]. In addition, given the high consanguineous rate in our pedigree and the fact that, due to costs and diagnostic setting no whole exome sequencing has been performed, a second underlying genetic condition responsible for the occipital defects in our cohort cannot be ruled out. Therefore it seems premature to include the failure of ossification of the occipital bone as a major diagnostic criterium yet. Future reports on skull defects in MADA and MADB patients are required to further underline that occipital ossification defects might be regarded a pathognomonic sign in MADB patients.

In contrast to the patient reported by Haye et al. [6], the patients in our cohort did not have gaze palsy and ptosis was noted in only one patient. Gaze palsy and ptosis might thus have been a coincidental finding in the patient described by Haye et al. and not a true clinical feature of MADB.

All of our patients had a normal psychomotor development. It has been suggested that the central nervous system is spared in patients with *LMNA*-linked disorders via downregulation of LMNA transcripts in the brain [22]. The *ZMPSTE24* gene mutated in MADB patients encodes the zinc metalloprotease enzyme involved in production of mature lamin A. We therefore hypothesize that downregulation of LMNA transcripts in the brain might also explain the lack of neurodevelopmental disabilities in MADB patients.

Our longitudinal cohort shows variable phenotypes across ages, and we observed that some clinical features of MADB developed over time. For example, our three youngest patients (examined at the ages of 3, 6 and 11 years, respectively) did not have joint contractures, proptosis, alopecia, or (marked) facial lipoatrophy. Still, those patients fulfilled the clinical diagnosis of MADB based on our proposed criteria of ≥4 major clinical features OR ≥ 3 major clinical features and ≥ 2 minor clinical features (Table 3). Only one previously published 9-month-old patient did not fulfil our MADB criteria [11]. He showed three major features: short stature, mandibular hypoplasia and skin abnormalities (i.e. atrophic and sclerodermic skin and mottled pigmentation). No minor features were present in this patient, indicating that in very young children the diagnosis might be missed. For instance, the major features delayed closure of cranial sutures and dental crowding cannot be assessed at a very young age. Thus re-evaluation should be performed when children grow older. However, the absence of several MADB features in the 9-month-old patient [11] might also be due to his limited clinical description. In a previously reported 10-month-old patient MADB could be diagnosed based on the presence of five major features, i.e. mandibular hypoplasia, clavicular hypoplasia, acro-osteolysis of distal phalanges, hypoplastic nails and skin abnormalities (atrophic skin and mottled pigmentation) and three minor features, i.e. lipoatrophy of the extremities, shortened phalanges and joint contractures [13]. Information on occipital ossification defects was, however, not available. It might also be that some publications may not have mentioned the presence or absence of certain features. Based on the current cohort and previously published MADB patients, we suggest that our MADB criteria can be used even in young patients, preferably from the age of 10 months onwards. These criteria will also help to evaluate if additional features, currently missing in the diagnostic criteria, need to be added, In addition more clinical data is needed to evaluate whether there is a genotype-phenotype correlation and if patients with MADB based on compound heterozygosity of a missense and a null mutation in the ZMPSTE24-gene may have a more severe phenotype than patients who have homozygous misssense mutations in this gene.

Children in low-income countries with limited access to genetic testing, in particular, could benefit from being diagnosed with MADB based on clinical criteria because it might guide clinical management. This is especially important because several complications are reported in MADB patients, including impaired glucose tolerance [23], diabetes mellitus [2, 23], glomerulopathy [1, 2] and vascular complications such as high blood pressure and atherosclerosis [2, 4]. In our population, patients complained about painful feet due to plantar atrophy of their feet. This can lead to wounds and, in combination with diabetes mellitus, might develop into extensive infections, making early diagnosis crucial in preventing these complications.

One of our patients died from heart failure. Since additional cardiological evaluation or autopsy was not performed, we can only speculate about the underlying cause. Important to mention is that there were no signs of renal failure. Patient VII is suffering from symptoms associated with cardiac failure. He is awaiting cardiac evaluation. Myocardial pathology has not been reported before in MADB patients [16]. Dilated cardiomyopathy has however been reported in ZMPSTE24-deficient mice [24–26]. Whether the heart failure in our patient is a solitary finding or this should be regarded part of mandibuloacral dysplasia requires further reports on MADB patients.

One of the limitations of our study is that additional investigations such as measuring blood and urine samples or performing X-rays could not be performed in most patients due to the limited availability of medical facilities. Secondly, the cause of death in one patient was uncertain but probably due to cardiac failure. Finally, although our clinical diagnostic criteria apply to most MADB patients they might not detect all MADB patients, especially those who are very young. Close follow-up of patients suspected of MADB, is therefore warranted.

## Conclusions

In this study, we report on eight related Surinamese patients with MADB due to a homozygous founder mutation in the *ZMPSTE24* gene. Our data illustrate the full phenotypic spectrum of MADB associated with a homozygous *ZMPSTE24* mutation. We propose clinical criteria for MADB that may be of use in settings with limited or no access to genetic testing. Future clinical research in MADB patients is needed to further refine our proposed clinical criteria because early diagnosis is essential for guiding clinical management and for family counselling.

## Data Availability

The datasets used and/or analysed during this study are available from the corresponding author upon request.

## Abbreviations

HGSP: Hutchinson-Gilford Progeria Syndrome
MADA: Mandibuloacral Dysplasia with type A lipodystrophy
MADB: Mandibuloacral Dysplasia with type B lipodystrophy
RD: Restrictive Dermopathy
WHO: World Health Organization
